# Supplementary material for: Description of Baseline Nutrition and Physical Activity Knowledge and Behavior in Acute Stroke/TIA Patients Enrolled in the Health Education on Information Retention and Behavior Change in Stroke (HERBS) Pilot Trial
Source: Nutrients. 2023 Aug 28;15(17):3761. doi: 10.3390/nu15173761 (PMC10490333; doi:10.3390/nu15173761)
Supplement: Supplementary file 1 [file nutrients-15-03761-s001.zip › nutrients-2541457-supplementary.pdf]

Supplementary Materials

Supplement S1. *Questionnaire.*

The percentage of respondents selecting each answer choice is parenthetically stated and correct answers are in boldened font. Response rates for each question are in parentheses after the question stem.

Physical Activity Knowledge

1. The American Heart Association recommends getting *at least* ___ minutes per week of moderate-intensity aerobic activity.
(96.2% response rate)

- **a. 150 minutes (29.6%)**
- b. 120 minutes (40.7%)
- c. 200 minutes (7.4%)
- d. 10 minutes (18.5)

2. The American Heart Association recommends muscle-strengthening activity (like resistance or weight training) *at least* ____ time(s) per week.
(96.2% response rate)

- a. 1 (14.8%)
- **b. 2 (29.6%)**
- c. 4 (37.0%)
- d. 7 (14.8%)

Nutrition Knowledge

1. At least how many servings of vegetables should you eat each day?
(100% response rate)

- a. 1 serving (0.5 cup or palm) (18.5%)
- b. 2 servings (1 cup or 1 fist) (44.5%)
- **c. 5 servings (2.5 cups or 2.5 fists) (33.3%)**
- d. 7 servings (3.5 cups or 3.5 fists) (3.7%)

2. At least how many servings of fruits should you eat each day?
(96.2% response rate)

- a. 1 serving (0.5 cup or palm) (18.5%)
- b. 2 serving (1 cup or 1 fist) (40.7%)
- **c. 4 serving (2 cups or 2 fists) (37%)**
- d. 8 serving (4 cups or 4 fists) (0%)

3. At least how many servings of whole grains should you eat each day?
(96.3% response rate)

- **a. 3-6 servings (3-6 ounces or 3-6 handfuls) (11.1%)**
- b. 1-2 servings (1-2 ounces or 1-2 handfuls) (81.5%)
- c. 6-8 servings (6-8 ounces o 6-8 handfuls) (3.7%)
- d. 0 servings (0 ounces or 0 handfuls) (0%)

4. Which of the below is an example of a whole grain?
(81.5% response rate)

- a. Brown rice (14.8%)
- b. Oatmeal (22.2%)
- c. Whole wheat pasta (7.4%)
- d. White bread (3.7%)
- **e. A, B, and C (33.3%)**

5. The American Heart Association recommends limiting added sugars to no more than
(88.9% response rate)

- a. 150 calories a day (9 teaspoons or 9 marbles) for women and 250 calories a day (15 teaspoons or 15 marbles) for men (25.9%)
- b. 200 calories a day (12 teaspoons or 12 marbles) for women and 150 calories a day (9 teaspoons or 9 marbles) for men (3.7%)
- c. 500 calories a day (30 teaspoons or 30 marbles) for women and 600 calories a day (36 teaspoons or 36 marbles) for men (0%)
- **d. 100 calories a day (6 teaspoons or 6 marbles) for women and 150 calories a day (9 teaspoons or 9 marbles) for men (59.3%)**

6. According to the American Heart Association, what is the biggest source of added sugar in the American diet?
(85.2% response rate)

- **a. Sugar-sweetened beverages (55.6%)**
- b. Fruit (3.7%)
- c. 100% Fruit Juice (14.8%)
- d. Ice Cream (11.1%)

7. The American Heart Association recommends eating no more than ______ of sodium in a day.
(92.6% response rate)

- a. 3500 mg (14.8%)
- **b. 1500 mg (29.6%)**
- c. 1000 mg (48.1%)
- d. 5000 mg (0%)

8. Too much sodium/salt may put you at risk for:
(88.9% response rate)

- a. Stroke (11.1%)
- b. Heart failure (0%)
- c. Kidney stones (3.7%)
- **d. All of the above (63%)**
- e. None of the above (11.1%)

9. Sodium is often “hidden” in which of the below?
(100% response rate)

- a. Soup (3.7%)
- b. Restaurant foods (3.7%)
- c. Processed foods (22.2%)
- **d. All of the above (70.4%)**

10. __________ fat lower rates of heart disease and all-cause death.
(100% response rate)

- **a. Unsaturated (18.5%)**
- b. Saturated (14.8%)
- c. Trans (22.2%)
- d. All of the above (44.5%)

11. Which of the below are unsaturated fat(s)? **Circle all that apply.**
(100% response rate)

- **a. Fish (55.6%)**
- **b. Nuts (59.3%)**
- c. Red meat (29.6%)
- d. Butter (29.6%)
- **e. Avocado (40.7%)**
- f. Heavy Cream (22.2)
- g. Cheese (33.3%)
- **h. Olive oil (55.6%)**

12. ___________ fat raises the risk of developing heart disease and raises bad cholesterol (LDL) levels.
(96.3% response rate)

- a. Unsaturated (3.7%)
- **b. Saturated (25.9%)**
- **c. Trans (11.1%)**
- d. All of the above (55.6%)

13. One cup of food is roughly equal to the size of
(96.3% response rate)

- **a. A fist or a baseball (51.9%)**
- b. A palm or a tennis ball (25.9%)
- c. Two fists or a softball (3.7%)
- d. A thumb or a ping pong ball (14.8%)

14. Three ounces of meat is roughly equal to the size of
(100% response rate)

- a. A thumb or a ping pong ball (18.5%)
- **b. A palm or a decks of cards (48.2%)**
- c. A fist or a baseball (18.5%)
- d. Two palms or two decks of cards (14.8%)
